# Exploring Profiles and Variables Related to Burnout Amongst School Mental Health Providers

**DOI:** 10.3390/bs15091289

**Published:** 2025-09-21

**Authors:** Ashley Rila, Gerta Bardhoshi, Derek Rodgers, Allison Bruhn, Duhita Mahatmya

**Affiliations:** College of Education, The University of Iowa, Iowa City, IA 52242, USA

**Keywords:** burnout, school mental health providers, job satisfaction, organizational support, social emotional behavioral health training, self-compassion, coping ability

## Abstract

The purpose of this cross-sectional study was to examine burnout profiles and organizational variables that impact burnout in school mental health providers, such as school counselors, school psychologists, social workers, and interventionists. We disseminated a survey to a large sample of school personnel across a Midwestern state. From the larger sample, we analyzed responses from school mental health providers (n = 120), as there are severe shortages of these professionals within the state and across the country. When shortages occur, caseloads are higher, thus increasing the work demands and the propensity for burnout. With burnout leading to attrition from the field, this creates a vicious cycle that could be prevented through the awareness and implementation of strategies to counteract the factors contributing to burnout. Results indicate school mental health providers (n = 120) in this state endure high levels of emotional exhaustion, while still maintaining a sense of personal accomplishment. Further, higher levels of perceived organizational support and job satisfaction appeared to lower burnout, whereas respondents who worked in schools implementing a multi-tiered system of support experienced higher burnout. Key findings, implications for practice, limitations, and future directions are discussed.

## 1. Exploring Profiles and Variables Related to Burnout Amongst School Mental Health Providers

Given reports of heightened behavioral and mental health issues in K-12 schools post-pandemic, there has been an acute focus on student well-being over the last several years. Clear evidence of this can be seen in the federally required “return-to-learn” plans submitted by each state as a contingency for receiving Elementary and Secondary School Emergency Relief funds. Not only did these plans indicate ways schools would mitigate learning loss, but they also contained strategies to meet students’ increased social, emotional, and behavioral (SEB) needs, including an increase in providing school-based mental health services (State et al., 2024). While providing instruction and intervention that effectively attends to students’ mental health needs is critical, a related urgent need is ensuring the mental health and well-being of school mental health providers (SMHPs; e.g., counselors, psychologists, social workers), as these professionals are primarily responsible for attending to students with the most significant mental health needs. Both during and post-pandemic, SMHPs’ demands increased as more students required mental health services, and SMHPs were often responding through virtual meetings, phone calls, and emails rather than in-person (Hirsch et al., 2022). A national survey of nearly 500 educators and SMHPs found that as the pandemic unfolded and these professionals were isolated from their colleagues and students, they did not feel they were able to adequately meet their students’ social, emotional, and behavioral needs (Bruhn et al., 2022). Research has shown that when demands are high and resources are low, self-efficacy wanes (Benight et al., 1999) and results in emotional exhaustion (Brunsting et al., 2014). Presumably, this was exacerbated during the pandemic. While returning to in-person instruction has eliminated the isolation and SMHPs have greater access to resources in their schools, it has not curtailed the demand for services. Unsurprisingly, SMHPs are highly susceptible to burnout, which may lead to attrition from the field (Bardhoshi et al., 2014; Bardhoshi & Um, 2021). In this paper, we examine the burnout profiles of SMHPs in one Midwestern state, as well as the extent to which systems-level, or organizational, variables (i.e., organizational support, professional development, multi-tiered systems of support implementation) impact burnout.

### 1.1. Burnout of SMHPs

The recent literature on SMHPs, including school counselors, school psychologists, and school social workers, indicates these professionals report high levels of workplace stress and widespread experiences of burnout (Bardhoshi & Um, 2021; Carnes, 2023; Pike et al., 2025; E. J. Schilling et al., 2018). Qualitative studies specifically situated in the context of the COVID-19 pandemic indicate that SMHPs consistently report being “overworked”, “overwhelmed” and “stretched thin” and experiencing “emotional exhaustion” and “compassion fatigue” (Bardhoshi et al., 2022; Carnes, 2023). While burnout is classified by the World Health Organization (2023) as an occupational phenomenon, research on SMHP burnout highlights its emergence both at the individual level and within the broader context of the school environment. On a personal level, burnout often manifests as emotional exhaustion, a reduced sense of professional accomplishment, and emotional detachment from students (Maslach et al., 2001). Beyond simply feeling overwhelmed, occupational burnout is linked to numerous adverse physical and mental health outcomes, including chronic fatigue, sleep disturbances, an increased risk of cardiovascular disease, substance use, and weakened immunity (Armon et al., 2008; Melamed et al., 2006; Peterson et al., 2008). At the organizational level, burnout is shaped by perceptions of a negative workplace climate, with adverse effects extending into professionals’ personal lives (S. M. Lee et al., 2007). As such, addressing burnout in SMHPs requires a comprehensive understanding of both intra-individual and systemic contributors.

Exploring the unique training and professional experiences that shape SMHP roles is also critical, as these experiences can act as either buffers or contributors to burnout. In response to this complexity, researchers have increasingly focused on identifying distinct burnout profiles and related psychological constructs in SMHPs (Bardhoshi et al., 2022; Ko & Lee, 2021; S. M. Lee et al., 2010). Burnout profiles classify mental health providers based on clusters of scores across the core burnout dimensions, including emotional exhaustion, depersonalization, and personal accomplishment (Bardhoshi et al., 2022; I. Kim et al., 2024; S. M. Lee et al., 2010). In addition to classifying burnout patterns quantitatively, researchers have also conducted mixed-methods explorations to highlight the relevance of organizational-specific barriers and resources across burnout profiles (Bardhoshi et al., 2022).

Researchers have also highlighted the importance of personal factors and experiences for SMHP burnout, with constructs such as adaptive emotional coping, mindful self-care, self-compassion, and job satisfaction either being inversely related to burnout or moderating the effects of emotional exhaustion (Bardhoshi & Um, 2021; Eriksson et al., 2018; Pike et al., 2025). Conceptually, an individual’s coping ability is directly related to their level of helplessness, which is one of the key factors associated with burnout (Schaufeli & Buunk, 2004). Indeed, a more flexible and positive orientation for handling stressful events explained a significant amount of variance in counselor trainees’ burnout (I. Lee et al., 2018), while maladaptive coping has been associated with significantly higher levels of burnout in helping professionals (Moate et al., 2016), including child and youth care workers (Savicki, 2002). Similarly, a coping orientation composed of an exaggerated sense of personal responsibility and self-blaming is associated with higher levels of burnout, as it leads individuals to internalize stressors and magnify feelings of inadequacy and emotional exhaustion (Leiter & Maslach, 2009). Relatedly, self-compassion is associated across studies with low burnout among mental health providers and is often recommended as an effective approach for preventing mental health professional burnout (Crego et al., 2022). Finally, job satisfaction can ameliorate experiences of burnout specifically by enhancing school mental health providers’ sense of purpose and motivation, fostering positive workplace relationships, and increasing resilience to stressors (Bardhoshi et al., 2022; Fye et al., 2020). This person-centered approach of accounting for personal and intra-personal factors in school mental health provider burnout allows for a more nuanced understanding of this phenomenon and supports the development of tailored interventions (Bardhoshi et al., 2022; Dreison et al., 2018).

### 1.2. Organizational Variables

In addition to intra-individual factors that contribute to job satisfaction and burnout, systems-level factors (i.e., organizational variables) exert a substantial influence on SMHP well-being, and, importantly, retention. Retention is a particularly salient issue across the country, as the ratios of SMHPs to students are significantly higher than those recommended by professional organizations (e.g., National Association of School Psychologists, American Counselor Association). For instance, the state in which this study was conducted has a school counselor to student ratio of 400:1, but the recommended ratio is 250:1. Even worse, the ratio of school psychologists is 1700:1, while the recommended ratio is 600:1. Further, recent legislative changes have resulted in cuts to funding area education agencies that employee SMHPs. Reduced funding and SMHP shortages create larger caseloads and more stress, which ultimately can lead to burnout and further attrition, creating a vicious cycle (E. Schilling et al., 2021). As such, there is a critical need to retain SMHPs and ensure there are sufficient mental health services for students. To meet this need, school systems must secure adequate organizational support and professional development opportunities for SMHPs.

Ongoing professional development in SEB prevention and intervention is a key component of multi-tiered systems of support (MTSS). A MTSS is a prevention framework that helps guide how faculty and staff respond to students’ needs. Common to MTSS prevention models are (a) the use of evidence-based practices; (b) data-based decision-making within and across tiers; and (c) systems to support these practices (e.g., leadership team, technology, professional development; Bruhn & McDaniel, 2021). Only a handful of studies have examined the relation between MTSSs and adult well-being, with these studies focusing on educators rather than SMHPs. For example, Ross et al. (2012) surveyed 184 teachers across 40 elementary schools and found teachers in schools implementing the MTSS model Positive Behavior Interventions and Supports (PBIS) with fidelity had significantly lower levels of burnout. Conversely, Oakes et al. (2013) found that the implementation fidelity of their MTSS model (called CI3T) did not have a significant relation to burnout levels. Of note, however, is the research demonstrating a significantly positive impact of PBIS on school climates (Bradshaw et al., 2008, 2009). Presumably, an improved school climate may lead to more positive feelings professionally and, in turn, less burnout for all faculty and staff. Research on the relation between MTSSs and the burnout of SMHPs could expand the literature beyond the research already conducted with teachers.

Professional development, as stated earlier, is an element of effective MTSS implementation (Bruhn & McDaniel, 2021). However, even in schools without an MTSS in place, professional development is likely a requirement for employees who need continuing education credits to maintain certification or licensure. Research has shown effective professional development can lead to improved knowledge and skills and, in turn, higher levels of self-efficacy (Bruhn et al., 2023), as well as increased job satisfaction (Darling-Hammond et al., 2017). This is particularly important given the relation between low self-efficacy and burnout amongst SMHPs (O’Brennan et al., 2017). If SMHPs have access to high-quality professional development that fills knowledge gaps and builds skills, their self-efficacy is likely to increase. This continuous improvement helps SMHPs meet the demands of the job, thus reducing stress while building feelings of competence.

A third factor shaping the job experience of SMHPs is organizational support. Broadly defined, organizational support refers to the extent to which school leaders (e.g., administrators), colleagues, and institutional policies demonstrate care and appreciation for employees. When SMHPs feel supported by their organization, they believe the organization (e.g., school) values their contributions and cares for their well-being (Xu & Yang, 2021). Some researchers have suggested that perceived organizational support (POS) can buffer the impacts of job stress. Specifically, Xu and Yang (2021) hypothesized that individuals who feel respected and cared for are likely to feel better about themselves and, thus, are better able to cope with job stress. Indeed, a lack of organizational support has been found to directly predict burnout in school counselors (Bardhoshi & Um, 2021), with organizational-specific barriers being directly cited by novice SMHPs as driving their burnout (Bardhoshi et al., 2022). On the other hand, social and emotional support at work can serve as a protective factor when job demands are high and resources are limited (Bakker & Demerouti, 2007; Bardhoshi et al., 2014; Bardhoshi & Um, 2021). This support may come from co-workers or supervisors. Principals, for example, play an important role in how organizational support is perceived, particularly by school counselors (Bardhoshi et al., 2014).

### 1.3. Purpose

SMHPs consistently have caseloads far beyond the recommended numbers, while they frequently serve students with the most significant mental health needs. Thus, it is imperative that school districts understand how to best support SMHPs personally and professionally. This includes identifying, preventing, and responding to feelings of burnout. To this end, our cross-sectional study extends the previous research on burnout by focusing on the burnout profiles of SMHPs and the organizational variables impacting burnout. Research questions include the following: (1) What are the burnout profiles of school mental health providers? (2) To what extent do organizational variables explain the variation in burnout amongst school mental health providers?

## 2. Method

### 2.1. Participants

The participants in this study represent a sub-sample from a larger group of school personnel, for whom data were collected for a larger project on school-based burnout conducted in a large, Midwestern state. We recruited participants in three distinct ways. First, we sent four emails to 24,000 K-12 educators. The email addresses were secured through a (a) publicly available email database published on the state’s Department of Education website, (b) purchased email database, and (c) university center’s email database. Four recruitment emails were sent between February and April 2024. Second, the recruitment emails requested the recipient to pass along the email to their fellow K-12 colleagues. Finally, we advertised the survey in three monthly newsletters for a university center during the same months the emails were sent. A link to the informed consent and survey instrument (described below) was included in each recruitment method. Of particular interest in this study are SMHPs, which we defined to include school counselors, behavioral specialists and interventionists, school psychologists, social workers, student–family advocates, and social–emotional learning specialists. One hundred sixty-four SMHPs completed at least some portion of the survey instrument. Some responders did not complete enough of the survey items to calculate the total and composite scores necessary for our analyses. Thus, our analytic sample was composed of the 120 individuals for whom we had the required information to calculate total and composite scores. Most participants were female (88.3%), White (97.5%), and had Master’s Degrees (88.3%). Participants averaged 45.2 years of age (SD = 10.6; Range = 22–63) and averaged 15.5 years of experience (SD = 5.9; Range = 2–21). More detailed participant demographics are presented in Table 1.

### 2.2. Survey Instrument

The survey instrument was designed to collect cross-sectional, quantitative data on several mental health-related constructs, including burnout, access to social–emotional–behavioral health (SEBH) training, providers’ sense of organizational support, their coping ability, and their levels of self-compassion. We also collected demographic data. All data were collected via Qualtrics, and all measures are described in greater detail below.

#### 2.2.1. Participant Demographics and School Characteristics

We asked SMHPs to provide us with demographic information, including their sex, race/ethnicity, age, years of experience, their position within their school, and their level of education. We also asked respondents to indicate whether their school system uses a multi-tier system of support (MTSS) and whether their school offers social–emotional–behavioral health (SEBH) training. For those who indicated that their schools do offer SEBH professional development training, we asked them to indicate how many SEBH trainings they had attended in the past five years.

#### 2.2.2. Burnout

We used the Maslach Burnout Inventory for educators (MBI; Mind Garden, 2017) to measure three facets of school personnel burnout. The MBI is a 22-item instrument, which includes a series of statements related to various aspects of burnout (e.g., “I feel emotionally drained from my work.”). Participants respond to each item using a 0–6 Likert-style scale (0 = Never; 3 = A few times a month; 6 = Every day). Responses can be totaled into three burnout subscales: emotional exhaustion (nine items, α = 0.91), depersonalization (five items, α = 0.75), and personal accomplishment (eight items, α = 0.79). Emotional exhaustion refers to feeling emotionally and mentally drained from work, depersonalization refers to a person’s detachment from their work, and personal accomplishment refers to a person’s belief that they make meaningful contributions in their work. Each of the three subscales can be further divided into low, moderate, and high categories. For the emotional exhaustion subscale, values less than 17, between 17 and 29, and 30 or greater are considered low, moderate, and high levels of emotional exhaustion, respectively. For the depersonalization subscale, values less than 5, between 5 and 11, and 12 or greater are considered low, moderate, and high levels of depersonalization, respectively. For the personal accomplishment subscale, values less than 33, between 33 and 39, and 40 or greater were considered low, moderate, and high levels of personal accomplishment, respectively. When generating scores for these subscales, we did not reverse score the items that comprise the personal accomplishment subscale to facilitate interpretation. However, we also calculated participants’ total burnout scores (α = 0.86) for our second phase of data analysis, during which time we did reverse score the items corresponding to the personal accomplishment subscale such that all items had identical interpretations (i.e., larger values indicate higher levels of burnout).

#### 2.2.3. Organizational Support

We used the 8-item version of the Survey of Perceived Organizational Support (SPOS-8; Eisenberger et al., 1986) to measure SMHPs’ sense of organizational support. All items pertain to respondents’ perception that their workplace organization values and appreciates their efforts. Items are written as statements, and respondents indicate the extent to which they agree using a 7-point, Likert-style scale (1 = Totally Disagree; 4 = Neither Agree nor Disagree; 7 = Totally Agree). All relevant items were reverse-scored prior to analysis. We created a composite score by adding all eight items together (α = 0.93).

#### 2.2.4. Coping Ability

We used the Brief Resilient Coping Scale (BRCS; Sinclair & Wallston, 2004) to measure participants’ level of ability to cope. This brief, 4-item measure asks respondents to complete items related to the extent to which they seek creative solutions to difficult situations, believe they can control their reactions to difficulty, believe they can grow in positive ways while dealing with difficult situations, and actively look for ways to replace losses in life. The BRCS uses a 5-point Likert-style scale (1 = Does not describe me at all; 3 = Neutral; 5 = Describes me very well). We created a composite by adding together the values of the four items (α = 0.68).

#### 2.2.5. Self-Compassion

We used the 12-item Self-Compassion Scale, Short Form (SCS; Raes et al., 2011) to measure participants’ levels of self-compassion. The SCS items are a series of statements about how respondents handle feelings of inadequacy, emotionally respond to their own difficulties and failings, and the extent to which they fixate on difficult feelings or ideas. Participants indicate how often they experience each statement using a 5-point, Likert-style scale (1 = Almost Never; 5 = Almost Always). We created composite scores, per measure guidelines, by first calculating six subscales, then dividing each person’s sum of those subscales by the number of items in a subscale (e.g., items 1 and 2 would be added and divided by 2), and then finally summing all of the averages (α = 0.60).

#### 2.2.6. Job Satisfaction

To measure job satisfaction, we asked participants to complete a single item about their job satisfaction. The item was worded as such, “If I consider every aspect of my job, I am satisfied with my job.” The reporting scale was a five-point Likert-style scale (1 = Strongly disagree; 3 = Neutral; 5 = Strongly Agree).

### 2.3. Data Analysis

Data were analyzed in two ways, each corresponding to one of the research questions. Regarding burnout profiles (Research Question 1), we analyzed the burnout, coping, self-compassion, and organizational support data descriptively. The burnout data were summarized by the three composites: emotional exhaustion, depersonalization, and personal accomplishment. The burnout, coping, and self-compassion instruments all include cutoffs for low, moderate, and high score levels, and the data were summarized in this framework. Our second research question focused on the extent to which organizational variables (organizational support, SMHPs’ attendance at SEBH trainings, and their schools’ use of an MTSS model) explain variation in responders’ total burnout scores. We also hypothesized that these associations might vary based upon SMHPs’ levels of coping and self-compassion, as well as their overall job satisfaction. Thus, we ran two multiple regression models. The first model examined the variation explained in total burnout by organizational variables. In this model, our dependent variable was participants’ total burnout scores, and our independent variables were the SPOS-8, participants’ attendance at SEBH training, and use of an MTSS model. The second model replicated the first while adding in SMHPs’ levels of coping, self-compassion, and job satisfaction as additional control variables. In addition to examining the strength and significance of the coefficients, we also examined changes in the *R*^2^ values between the two models.

All data analyses were conducted using RStudio (Version 2025.5.1.513) (R Core Team, 2022), primarily with the *lmtest* (Zeileis & Hothorn, 2002) and *psych* (Revelle, 2022) packages. Prior to data analysis, all continuous variables were centered and examined for collinearity. Broadly, we observed no problematic correlations between variables. The job satisfaction variable was strongly and negatively correlated with the total burnout variable (*r* = −0.69; see Table 2), but this was both expected and below common thresholds for collinearity.

## 3. Results

### 3.1. Burnout Profiles of School Mental Health Providers

The data analysis indicated that participants exhibited significant levels of burnout (complete data on participants’ burnout profiles are provided in Table 3). Specifically, nearly two-thirds of responders (64.2%) reported high levels of emotional exhaustion. Most SMHPs were split between low (38.3%) and moderate (39.2%) levels of depersonalization, and most SMHPs reported high levels of personal accomplishment (55.0%).

Although few SMHPs reported high levels of self-compassion (1.6%), the vast majority (76.7%) fell within the moderate range. Less than one-quarter of the responders (21.7%) had low levels of self-compassion. Similarly, SMHPs reported moderate-to-high levels of coping abilities. Nearly two-thirds of the sample (64.2%) reported high coping abilities, while another 25% reported moderate levels.

Participants reported mild to moderate levels of organizational support, averaging 31.5 (SD = 12.2) on the SPOS-8. Individual responses showed considerable variations, with observed SPOS-8 total scores ranging from 8 to 56, which is the range of possible values. The observed average is slightly above the center of the SPOS-8 scale (24).

### 3.2. Organizational Variables and Burnout

To answer our second research question, we ran two multiple regression models. The first model (Model 1A) evaluated the extent to which organizational variables (independent variables) explain variation in SMHPs’ total burnout, and the second model (Model 1B) evaluated the same associations with SMHPs’ level of coping, self-compassion, and job satisfaction added as control variables. The full results for these models can be found in Table 4.

The intercept for Model 1A was statistically significant, and the coefficient of 57.87 indicates that—at average, or centered, levels of SPOS-8 and the number of SEBH training courses attended by SMHPs and for SMHPs whose schools do not use an MTSS—the average total burnout score was just below 58, which is a mild to moderate value for the total burnout score, which can range from 0 to 132. The remaining results of Model 1A yield a single significant independent variable: the providers’ sense of organizational support, measured by the SPOS-8. The statistically significant coefficient indicates that for every unit increase in providers’ sense of organizational support, there is a corresponding decrease in total burnout of 0.48. Stated more succinctly, while controlling for the number of SEBH training courses SMHPs attended and whether their school implements some type of MTSS, SMHPs with a greater sense of organizational support had significantly lower levels of burnout. Neither the number of SEBH training sessions the SMHPs attended nor whether their school used an MTSS were statistically significant. The adjusted *R*^2^ value was 0.11, indicating that the explanatory variables explained 11% of the variation in the outcome.

The intercept for Model 1B was also statistically significant—the coefficient of which indicates that—at average, or centered, levels of SMHPs’ organizational support, number of SEBH training sessions attended, coping ability, self-compassion, and job satisfaction, and for SMHPs from schools that do not use an MTSS—the average total burnout was 96. Larger than the intercept for Model 1A, the newly included additional control variables (providers’ coping ability, level of self-compassion, and job satisfaction) had a considerable impact on the model results. This pattern is observed in the coefficient estimates. With the addition of the control variables, SMHPs’ sense of organizational support was no longer statistically significant. Interestingly, the MTSS status of the SMHPs’ schools became statistically significant in the second model, indicating that providers who work at schools with an MTSS reported, on average, higher levels of total burnout. Finally, SMHPs’ job satisfaction was also statistically significant. The coefficient indicates that for every unit increase in SMHPs’ job satisfaction, they experience a 9.80 unit decrease in total burnout—a considerable reduction. Stated another way, SMHPs with greater levels of job satisfaction had significantly lower levels of burnout. The *R*^2^ value of Model 1B increased to 0.51, meaning that the variables in the model account for over 50% of the variation in total burnout scores.

## 4. Discussion

The purpose of this study was two-fold: first, to broadly examine burnout profiles among SMHPs, and, second, to explore the relationship between organizational variables and burnout among a sample of SMHPs from a Midwestern state. Our descriptive results (Research Question 1) revealed that SMHPs in this study experienced significant levels of emotional exhaustion. Specifically, 64.2% of the SMHPs reported high emotional exhaustion, a key facet of burnout. These findings parallel findings from previous research (e.g., Bardhoshi & Um, 2021; Carnes, 2023). In contrast, depersonalization levels were more evenly distributed, with most SMHPs reporting low (38.3%) or moderate (39.2%) levels. Further, the majority of the SMHPs (55.0%) reported high levels of personal accomplishment. Collectively, these results suggest that despite elevated emotional exhaustion, many of the SMHPs in this sample continue to find meaning and purpose in their roles, and most have not yet reached the negative detachment phase (depersonalization). This echoes previous findings regarding school counselors, who appear to maintain high levels of positive regard and empathy for students despite high levels of emotional exhaustion (Bardhoshi et al., 2022; Gnilka et al., 2015). Relatedly, other researchers have found school psychologists are much more likely to rate themselves high on emotional exhaustion than on depersonalization, indicating that despite frustrations, they are still demonstrating a strong level of care for their students (e.g., E. J. Schilling et al., 2018).

Additionally, most of the SMHPs reported moderate self-compassion levels (76.6%) and moderate (25%) to high (64.2%) coping abilities, suggesting that the SMHPs in this study have adaptive strategies to handle the stressors and challenges they face in their work. While the presence of low depersonalization, high levels of personal accomplishment, moderate levels of self-compassion, and high levels of coping is encouraging, the high rates of emotional exhaustion should not be overlooked. These findings could be reflective of the context of the pandemic, as the rapid increase in demands and student crises and rapid online adaptation might have promoted a resilient approach in the midst of a crisis, often referred to as post-traumatic growth (Goodman-Scott & Perez, 2023). However, a decline in resources and disrupted routines could have increased stress levels among school mental health professionals (Litam et al., 2021), leading to high rates of emotional exhaustion. Although burnout is not necessarily linear (Maslach, 2003), these findings suggest a risk of progression toward severe burnout symptoms if mitigation strategies are not implemented. Without intervention, the current levels of elevated emotional exhaustion could evolve into increased depersonalization and diminished personal accomplishment. This pattern is often common in the context of a crisis, such as a pandemic—individuals may sustain energy and maintain resilience during the immediate crisis due to urgency and heightened purpose, but once the crisis subsides, the accumulated stress often requires time for reflection, processing, and support to prevent long-term burnout and a decline in well-being (Goodman-Scott & Perez, 2023; Litam et al., 2021). This is a serious consideration for the field, as addressing burnout is essential for retaining SMHPs in the aftermath of the COVID pandemic, a pressing concern raised by professional organizations such as the National Association of School Psychologists and the American Counselor Association.

While descriptive results provide a snapshot of the overall sample, the regression analyses (Research Question 2) offered additional insight into how organizational and individual factors predict burnout. In the first model, which included the perceived organizational support, SEBH training attendance, and MTSS status, only perceived organizational support emerged as a statistically significant predictor of burnout. Specifically, higher levels of perceived organizational support were associated with lower burnout scores, which is consistent with previous research (Xu & Yang, 2021). Given that the model accounted for 11% of the variance in burnout, this model suggests a modest but meaningful relationship between organizational support and SMHP well-being.

Model two provided an expanded relationship by introducing coping ability, self-compassion, and job satisfaction as additional predictors of burnout. The inclusion of these variables significantly improved the model fit, explaining 51% of the variance in burnout. Within this expanded model, job satisfaction emerged as a strong and statistically significant predictor of burnout, while perceived organizational support was no longer significant. Additionally, schools implementing an MTSS became statistically significant, wherein SMHPs working in non-MTSS schools reported higher burnout. Coping ability and self-compassion, though theoretically relevant, were not statistically significant.

The comparison between the two models highlights important shifts in the predictors of SMHP burnout when individual and organizational factors are considered. As seen in the first model and prior research (Bardhoshi & Um, 2021; Bakker & Demerouti, 2007; Xu & Yang, 2021), perceived organization support emerged as significant among SMHPs who felt valued and supported by their schools, and the SMHPs reported lower levels of burnout. However, when coping ability, self-compassion, and job satisfaction were added in model two, organizational support was no longer a significant predictor of burnout. This suggests that the protective effect of perceived support may be mediated or masked by job satisfaction, which emerged as a significant predictor. These findings align with the existing research indicating that job satisfaction can serve as a critical buffer against burnout (Bardhoshi et al., 2022).

With these two models, an interesting shift occurred with the MTSS school implementation variable. While MTSS implementation was not statistically significant in the first model, it became statistically significant in the second model, albeit in the opposite direction we expected. This contradicts previous findings that suggest MTSS implementation reduces burnout (e.g., Ross et al., 2012) and instead more closely aligns with studies such as Oakes et al. (2013), which found no protective effect of MTSS implementation on burnout. This unexpected finding warrants further investigation, as it may reflect increased job demands, role ambiguity, and the additional stress associated with a MTSS, especially in SMHP contexts (Culbreth et al., 2005).

### 4.1. Implications for Practice

This study offers important implications for school mental health providers (SMHPs)—including school counselors, social workers, psychologists, and related professionals—as well as school administrators and researchers, focusing on preventing burnout, promoting well-being, and sustaining professional engagement. The elevated emotional exhaustion observed among SMHPs highlights the critical need for strategies that support their mental health and professional sustainability. Encouragingly, many SMHPs maintain strong personal accomplishment and moderate levels of self-compassion and coping, indicating positive adaptation strategies that can be strengthened to address burnout (Pike et al., 2025; Um & Bardhoshi, 2024). For example, educators and trainers preparing SMHPs should integrate wellness-focused content—such as self-compassion training, stress management, and burnout awareness—into professional preparation and ongoing development. This professional development content can be used to enhance critical career-sustaining behaviors that enhance job satisfaction, help ameliorate work challenges (Lawson & Myers, 2011), and can be essential in addressing burnout (Mullen et al., 2018). Creating spaces for open dialog about work-related stress and burnout in both training and practice settings can normalize these challenges and foster peer support networks across disciplines. Emphasizing holistic professional identity development and well-being across all SMHP roles is essential for sustaining the workforce.

The findings emphasize that both perceived organizational support and job satisfaction are key factors impacting burnout. While support from leadership is important, job satisfaction emerged as a stronger predictor of lower burnout, signaling the necessity of fostering work environments where SMHPs feel valued, respected, and fulfilled. Previous findings have reported that burnout can explain up to 40% of the variance in school counselor job satisfaction, highlighting the importance of strategically addressing SMHPs’ individual work experiences (Mullen et al., 2018). Administrators should prioritize clear role definitions—especially within complex frameworks like MTSSs—and workload management to mitigate role ambiguity and prevent stress accumulation. Indeed, role ambiguity stemming from conflicting and unclear expectations about professional responsibilities has been associated with reduced job satisfaction and elevated burnout among mental health providers, including school counselors and social workers (Bardhoshi et al., 2014; Culbreth et al., 2005; H. Kim & Stoner, 2008; Mullen et al., 2018). The unexpected association between MTSS implementation and higher burnout rates among SMHPs calls for careful attention to how these systems are deployed. For instance, ensuring SMHPs have adequate training, resources, and role clarity around the MTSS may help avoid inadvertently increasing the burnout risk.

These findings underscore the need for continued investigation into the multilevel factors influencing burnout across diverse SMHP roles. Future studies should explore how job satisfaction mediates the relationship between organizational support and burnout and further unpack the impact of MTSSs and similar systemic initiatives on SMHP well-being. Longitudinal and mixed-methods research designs are recommended to better understand the evolving nature of professional identity and occupational health in SMHP populations. Additionally, intervention research focusing on both individual resilience-building (e.g., adaptive emotional coping, mindful self-care) and organizational change is vital for developing effective strategies to enhance SMHP retention and engagement and reduce burnout (Dreison et al., 2018). Together, these implications advocate for a comprehensive, multi-pronged approach involving professional preparation, supportive organizational climates, and rigorous research to address burnout and promote sustainable careers for all school mental health providers.

### 4.2. Limitations and Future Directions

Several limitations should be considered when interpreting the findings of this study. First, the anonymous, self-report nature of this survey may have introduced bias. The participants may have under- or over-reported their experiences due to social desirability or recall challenges. It is also possible that, though likely infrequent, a participant may have submitted a survey more than once, which may have impacted data integrity. Given the anonymous nature of the project, participants may not have understood all of the questions accurately. For example, 16 participants stated they did not work in an MTSS school; this is unlikely given that most schools in this Midwestern state implement an MTSS in some capacity. Similarly, participants may not have accurately recognized whether they had attended SEBH training sessions, as SEBH encompasses a wide range of topics that may not have been explicitly labeled as such. Furthermore, we were unable to capture the MTSS implementation fidelity or the specific responsibilities of the SMHPs within an MTSS framework. These contextual factors may explain why SMHPs in MTSS schools reported higher burnout, as the fidelity of the MTSS implementation has been found to affect burnout (e.g., Oakes et al., 2013). Future researchers should capture contextual factors, such as school-level MTSS implementation data or the SEBH training provided to faculty and staff, to enable the field to better understand SMHP burnout. One way to obtain these data would be through partnering with school districts and asking participants to report their specific MTSS role. Additionally, to improve validity, future researchers should triangulate additional data sources, such as administrative records, peer or supervisor ratings, and observations of workplace practices.

The timing of the data collection may have also influenced participant responses. The survey timing coincided with the state legislative cycle in which funding was cut for Area Education Agencies (AEAs). These AEAs historically provided critical regional support, such as professional development, special education services (e.g., school psychology, occupational therapy), and instructional resources, free of charge to schools. This legislative change introduced widespread uncertainty for many SMHPs, which may have amplified feelings of emotional exhaustion and impacted their perceptions of organizational support and job satisfaction. Future researchers should consider how policy and funding shifts affect burnout. Similarly, the cross-sectional design of this study complicates our ability to determine the directionality of the relationship between burnout and job satisfaction. These limitations could be addressed through longitudinal studies to capture experiences over time.

The format of some survey items could have impacted the variability and validity of the findings. The job satisfaction item was measured using only a single, five-point Likert-style scale item. Similarly, the SEBH and MTSS items—both dichotomous—revealed only whether respondents’ schools did or did not include those supports in their school systems. Using multiple items to capture the complexity of these factors may have yielded more nuanced results.

Finally, the participants in this study were overwhelmingly White (97.5%) and female (88.3%), which potentially limits the generalizability of these findings. While the sample may reflect the demographics of school mental health professionals (SMHPs) in the Midwestern state studied, no publicly available database tracks these demographics at the state level to our knowledge. That said, national data indicate that school psychologists, school counselors, and school social workers are 78.3%, 74%, and 60.5% White and 83.9%, 87%, and 83.5% female, respectively (American School Counselor Association, 2023; DataUSA, 2023). These data indicate that both nationally and within this study SMHPs indeed are disproportionally White and female. Nonetheless, future researchers and state education agencies are encouraged to collect and report demographic characteristics (e.g., race, gender) for school professionals to improve transparency and enhance the applicability of research findings.

## 5. Conclusions

In summary, this study explored the burnout profiles of SMHPs and the organizational variables that may explain the burnout variation. While findings revealed elevated emotional exhaustion, many providers maintained high levels of personal accomplishment and adaptive coping, underscoring their resilience and commitment despite significant stress. Importantly, job satisfaction emerged as a strong predictor of reduced burnout, outweighing the protective role of organizational support alone, and schools implementing an MTSS unexpectedly showed higher provider burnout. These results emphasize the need for systemic efforts that not only increase organizational support but also foster job satisfaction through role clarity, manageable workloads, and professional recognition. Future researchers should continue to explore how contextual and organizational factors intersect with individual coping strategies to better understand and mitigate burnout, which may help ensure the sustainability of the SMHP workforce.

## Figures and Tables

**Table 1:** Participant demographics.

Variable	n (%)	Variable	n (%)
Sex		Race	
Female	106 (88.3%)	White	117 (97.5%)
Male	13 (10.8%)	Other/Prefer Not to Say	3 (2.5%)
Other/Prefer Not to Say	1 (0.8%)		
		Level of Education	
Position		Bachelor’s Degree	5 (4.2%)
School Counselor	95 (79.2%)	Master’s Degree	106 (88.3%)
Behavior Specialist	5 (4.2%)	Education Specialist	6 (5.0%)
School Psychologist	7 (5.8%)	Doctoral	2 (1.7%)
Social Worker	6 (5.0%)	Not Reported	1 (0.8%)
Student-Family Advocate	2 (1.7%)		
Multiple Roles	5 (4.2%)	School Offers SEBH PD	
		Yes	75 (62.5%)
My School Uses MTSS		No	43 (35.8%)
Yes	102 (85.0%)	Not Reported	2 (1.7%)
No	16 (13.3%)		
Not Reported	2 (1.7%)		

*Note*. n = total number of participants identifying as this variable; % = percent of sample identifying as this variable; and SEBH PD = social–emotional–behavioral health professional development.

**Table 2:** Correlations between continuous predictor variables.

	SPOS Total	BRCS Total	SCS Total	Satisfaction	SEBH	BO Total
SPOS Total	1.0					
BRCS Total	0.17	1.0				
SCS Total	0.17	0.37	1.0			
Satisfaction	0.48	0.33	0.16	1.0		
SEBH	−0.02	0.24	0.03	−0.14	1.0	
BO Total	−0.35	−0.32	0.28	−0.69	0.09	1.0

*Note*. SPOS Total = total score on the Survey of Perceived Organizational Support, 8th edition; BRCS Total = total score on the Brief Resilient Coping Scale; SCS Total = total score on the Self-Compassion Scale; Satisfaction = job satisfaction score; SEBH = number of social–emotional–behavioral health training courses attended; and BO total = total score on the burnout measure.

**Table 3:** Percent of respondents classified with low, moderate, and high levels of burnout by category.

Burnout Type	Low	Moderate	High
Emotional Exhaustion	10.0%	25.8%	64.2%
Depersonalization	33.3%	39.2%	22.5%
Personal Accomplishment	11.7%	33.3%	55.0%

**Table 4:** Regression model results exploring variation in total burnout.

Variable	Est.	SE
Intercept	47.64 ***	3.72
SPOS-8 Total	−0.48 ***	0.12
Number of Training Courses Attended	0.06	0.71
MTSS	2.36	4.02
	*Adjusted R* ^2^	0.11
Intercept	96.00 ***	10.14
SPOS-8 Total	−0.04	0.10
Number of Training Courses Attended	−0.06	0.56
MTSS	7.17 *	3.07
Coping Ability	−0.25	0.57
Self-Compassion	−5.78	3.06
Job Satisfaction	−9.80 ***	1.16
	*Adjusted R* ^2^	0.51

*Note*. SEBH = social–emotional–behavioral health; SE = standard error; * = significant at >0.05; and *** significant at >0.001.

## Data Availability

The dataset generated and analyzed in this study is not publicly available.
